# The significance of glycolysis index and its correlations with immune infiltrates in Alzheimer’s disease

**DOI:** 10.3389/fimmu.2022.960906

**Published:** 2022-10-24

**Authors:** Zhiqiang Qiu, Xuanyang Bai, Xiangwen Ji, Xiang Wang, Xinye Han, Duo Wang, Fenjun Jiang, Yihua An

**Affiliations:** ^1^ Department of Neurosurgery, Sanbo Brain Hospital, Capital Medical University, Beijing, China; ^2^ School of Public Health, China Medical University, Shenyang, China; ^3^ Department of Biomedical Informatics, Department of Physiology and Pathophysiology, Center for Noncoding RNA Medicine, School of Basic Medical Sciences, Peking University, Beijing, China; ^4^ Department of Research and Development, Beijing Yihua Biotechnology Co., Ltd, Beijing, China

**Keywords:** Alzheimer’s disease, glycolysis index, brain cell markers, prognostic indicator, microglia

## Abstract

Alzheimer’s disease (AD) is a common neurodegenerative disorder without an effective treatment, and results in an increasingly serious health problem. However, its pathogenesis is complex and poorly understood. Nonetheless, the exact role of dysfunctional glucose metabolism in AD pathogenesis remains unclear. We screened 28 core glycolysis-related genes and introduced a novel metric, the glycolysis index, to estimate the activation of glycolysis. The glycolysis index was significantly lower in the AD group in four different brain regions (frontal cortex, FC; temporal cortex, TC; hippocampus, HP; and entorhinal cortex, EC) than that in the control group. Combined with differential expression and over-representation analyses, we determined the clinical and pathological relevance of glycolysis in AD. Subsequently, we investigated the role of glycolysis in the AD brain microenvironment. We developed a glycolysis-brain cell marker connection network, which revealed a close relationship between glycolysis and seven brain cell types, most of which presented abundant variants in AD. Using immunohistochemistry, we detected greater infiltrated microglia and higher expression of glycolysis-related microglia markers in the APP/PS1 AD model than that in the control group, consistent with our bioinformatic analysis results. Furthermore, the excellent predictive value of the glycolysis index has been verified in different populations. Overall, our present findings revealed the clinical and biological significance of glycolysis and the brain microenvironment in AD.

## Introduction

Alzheimer’s disease (AD) is one of the most common age-related neurodegenerative diseases, and is clinically characterized by progressive cognitive decline with memory impairment (1). Its symptoms usually begin with mild cognitive impairment (MCI). However, cognitive difficulties and dysfunction in complex daily activities occur with disease progression (2). The clinical diagnosis of AD presents with neuropathological changes, such as extracellular amyloid plaques composed of the amyloid β (Aβ) peptide, intraneuronal neurofibrillary tangles (NFTs) composed of hyperphosphorylated tau protein, the binding of Apolipoprotein E to Aβ, the activation of immune mediators (including reactive astrogliosis and microgliosis), and reductions in synaptic density (3, 4). According to epidemiological findings, AD was the seventh leading cause of death in the United States in 2020 and 2021 (5, 6), and is estimated to affect approximately 6.5 million Americans aged ≥65 years today. Compared with heart disease, stroke, and human immunodeficiency virus, whose death rate decreased from 2000 to 2019, deaths from AD reportedly increased by >145% (7). AD has become an escalating burden on the society; nonetheless, there is still no cure.

In recent years, the relationship between dysfunctional glucose metabolism and AD has attracted considerable attention (8–11). Under normal physiological conditions, the human brain constitutes only 2% to 3% of the body weight of adults; however, its metabolism accounts for 25% of whole-body glucose utilization (12, 13). The brain requires a high supply of energy, but has little capacity for energy production; thus, scientists have postulated the interesting “selfish brain theory” (14–16). Similar to other tissues, glucose is metabolized in the brain by classical pathways, including glycolysis, tricarboxylic acid cycle, hexose monophosphate shunt, and electron transport chain (17). Glucose metabolism is significantly impaired under the pathological conditions of AD. Using positron emission tomography, previous clinical studies demonstrated that cerebral glycolysis is reduced in individuals with early AD and positive Aβ, with or without MCI (8, 9, 18, 19). In addition, several clinical features of individuals with type 2 diabetes mellitus are closely related to AD, such as modest cognitive deficits, mood disorders, and cerebral atrophy (20–23). Intrinsic brain insulin resistance is one of the potential mechanisms underlying AD (10). At the molecular level, a growing number of glycolysis-related proteins are dysfunctional in AD. For instance, a redox proteomics study revealed that glycolytic enzymes, i.e., aldolase, α-enolase, triosephosphate isomerase (TPI), glyceraldehyde-3-phosphate dehydrogenase and phosphoglycerate mutase 1, undergo an oxidative modification in AD brain tissues (24). Another study demonstrated that bolstering NAD+/NADH using nicotinamide riboside, potentially *via* glycolysis, may contribute to AD treatment (25). These findings suggested that glycolysis may play an important role in AD; thus, we systematically aimed to investigate the clinical and biological significance of glycolysis in AD.

We aimed to demonstrate the wide downregulation of glycolysis in multiple brain regions of patients with AD. Meanwhile, we curated a glycolysis-related 28-gene set and designed a single-sample gene set enrichment analysis (ssGSEA)-based metric, the glycolysis index. Differentially expressed genes (DEGs) between normal and AD brain tissues and between AD samples with low and high glycolysis indices were selected for the over-representation analysis. Moreover, we investigated the relationship between glycolysis and the brain microenvironment in patients with AD. Using immunohistochemistry (IHC), we further confirmed our bioinformatic analysis results that there were greater infiltrated microglia and higher expression of glycolysis-related microglia marker Spalt Like Transcription Factor 1 (Sall1) in the APP/PS1 AD model than that in the control group at the protein level (26). Eventually, we verified the predictive performance of the glycolysis index in the normal population. Collectively, we demonstrated the clinical and biological significance of glycolysis in AD and presented potential targets for its interaction with the brain microenvironment.

## Methods and materials

### Data collection

We accessed normalized gene expression profiles and differential expression analysis results from four different brain parts (frontal cortex, FC; temporal cortex, TC; hippocampus, HP; and entorhinal cortex, EC) from postmortem donors with and without AD using AlzData (27) (http://www.alzdata.org/index.html) as well as their corresponding clinical information. Microarray data [GSE84422 (28)] of 2,004 brain samples with Braak neurofibrillary tangle scores were obtained from the Gene Expression Omnibus (GEO) datasets (https://www.ncbi.nlm.nih.gov/gds/) as a validation dataset, which was divided into three sub-datasets using different microarray platforms. We transformed the probes of different platforms into gene symbols using BioMart (29). In addition, we downloaded RNA-seq data of normal brain tissues in transcripts per kilobase per million mapped reads format from the Genotype-Tissue Expression project (30) (https://gtexportal.org/home/) v8 data. Duplicate gene symbols were summarized using the median values.

### Glycolysis index calculation

We searched and downloaded seven glycolysis-related gene sets (glycolytic process through fructose-6-phosphate, REACTOME: glycolysis, Kyoto Encyclopedia of Genes and Genomes (KEGG): glycolysis gluconeogenesis, BIOCARTA: glycolysis pathway, HALLMARK: glycolysis, WP: glycolysis and gluconeogenesis, and WP: glycolysis in senescence) from the Molecular Signatures Database (http://www.gsea-msigdb.org/gsea/msigdb/index.jsp) (31). After applying GSEA to four AlzData datasets using the seven gene sets, we selected the 28 leading genes in at least half (two) of the datasets as the core glycolysis genes. The glycolysis index was defined as the enrichment score (ES) of the ssGSEA algorithm using the core glycolysis genes. GSEA and ssGSEA were performed using the R (v4.1.0) package clusterProfiler (32) (v4.0.5) and Python (v3.9.1) package gseapy (v0.10.2), respectively.

### Differential expression analysis

We performed differential expression analyses between normal and AD brain tissues, and between AD samples with low and high glycolysis indices using the R package limma (33) (v3.48.3). We combined the four parts of AlzData datasets for the analyses. Parts of the brain, age, and sex were included as the covariates. The different groups are divided by the median values of glycolysis index into different brain regions, respectively. As the continuous values, ages were converted to natural cubic splines with one degree of freedom according to the user guide of the limma.

### Over-representation analysis

We performed gene ontology (GO) and KEGG enrichment analyses using the R package clusterProfiler (32) (v4.0.5). Gene symbols were transformed as the Entrez gene ID before analyses using the R package org.Hs.eg.db (v3.13.0). Genes that existed in all four AlzData datasets were used as the background genes.

### Brain cell markers/signatures collection

The marker genes of several brain cells, including astrocytes, endothelial cells, microglia, neural stem cells, neurons, oligodendrocytes, oligodendrocyte progenitor cells (OPC), and Purkinje cells, were obtained from the CellMarker (34) database (http://biocc.hrbmu.edu.cn/CellMarker/index.jsp). We obtained a brain single-cell RNA-seq dataset [GSE67835 (35)] from GEO datasets, which contains six brain cell types (astrocytes, endothelial cells, microglia, neurons, oligodendrocytes, and OPC) following filtration, and could be adopted as a signature matrix for cell abundance deconvolution.

### Cell abundance estimation

We used the proportion of immune and cancer cells [EPIC (36)], immune cell abundance identifier [ImmuCellAI (37)], and CIBERSORTx (38) to estimate the abundance of immune cells in AlzData samples. In addition, based on the previously mentioned brain single-cell sequencing data, we used CIBERSORTx for predicting custom brain cell abundance. EPIC was implemented using the R package EPIC (v1.1.5), and ImmuCellAI and CIBERSORTx were performed using their respective web tools. The file format was prepared in accordance with the guidelines of the corresponding tool. For CIBERSORTx, quantile normalization was disabled and the program was run in an absolute mode. As a supplement, we used ES to estimate the relative abundance of microglia in the sample using microglia marker genes and ssGSEA.

### Network construction

Separately, we calculated the expression correlations among genes from seven glycolysis-related gene sets and brain cell marker genes from CellMarker using four AlzData datasets. P-values were adjusted using the Benjamini and Hochberg method. Co-expression with a false discovery rate (FDR) <0.05 was considered significant, and we required it to have identical correlation directions in all datasets with significant results. In addition, we extracted the protein-protein interaction (PPI) information from the STRING (39) database, where high-confidence interactions with scores >0.7 were retained. We used gene pairs with significant/high-confidence results in at least four of the five metrics (four datasets and one interaction) for subsequent network construction and functional validation. The network was constructed using Cytoscape (40) (v3.8.2) in Java (v11.0.9.1).

### Immunohistochemistry

Specific pathogen-free male C57BL/6J mice and APP/PS1 mice (22-24 weeks old, 20–25 g) were purchased from the Changzhou Cavens Laboratory Animal Co., Ltd. (Jiangsu, China). The animals were subjected to adaptive feeding for 1 week. Following anesthesia, mice brains were fixed by cardiac perfusion with phosphate buffered saline (PBS), and subsequently with 4% paraformaldehyde (PFA). The brains were removed and stored overnight in 4% PFA at 4°C. The tissues were dehydrated using graded ethanol in xylene and embedded in paraffin wax. Sections (5μm) were cut in the paraffin sagittal plane and mounted on glass slides. We performed immunohistochemical staining on the sections following deparaffinisation. Paraffin sections were blocked against endogenous peroxidase activity with 3% H_2_O_2_ and subjected to antigen retrieval, followed by blocking. Subsequently, the sections were incubated overnight with antibodies against ionized calcium-binding adapter molecule 1 (Iba1) (1:1000, ab178846, Abcam) and Sall1 (1:200, ab41974, Abcam) at 4°C. The following day, the sections were washed with PBS, stained with secondary antibodies, developed with 3,3′-diaminobenzidine chromogenic solution, counterstained with hematoxylin, dehydrated, and mounted. Two observers reviewed and independently quantified the expression levels using the percentage of positive cells and the average optical density using Alpathwell software (Servicebio, Wuhan, China). All animal experimental protocols were approved by the Animal Experiments and Experimental Animal Welfare Committee of the Capital Medical University.

### Statistical analysis

We performed two-sided Wilcoxon rank-sum tests to evaluate the differences between the two groups of continuous values. The Spearman’s correlation test was conducted to calculate the significance of the correlations between the groups of continuous values. We used the analysis of variance (ANOVA) and linear models to test if the correlation between the two indicators was independent of other factors. All statistical analyses were performed using R (v4.1.0), Python (v3.9.1), and GraphPad Prism 9 (v9.0.0 for macOS). Statistical significance was set at P-values or FDR <0.05.

## Results

### The clinical relevance of glycolysis index in AD

**Figure 1:**
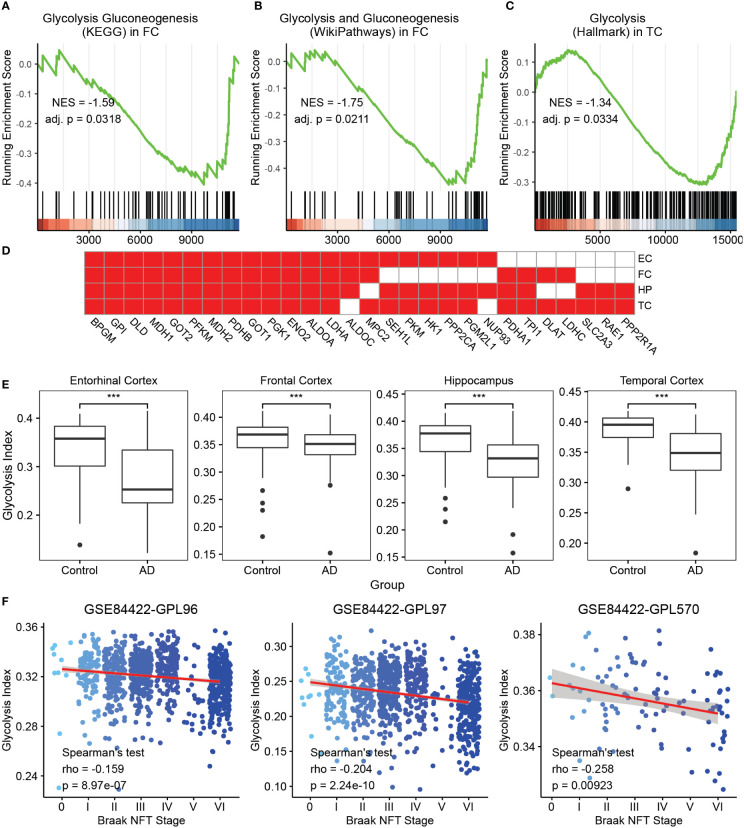
Glycolysis index and its clinical relevance in AD **(A-C)** GSEA analysis on AD vs. control samples of the glycolysis gluconeogenesis **(KEGG)** gene set in FC. **(A)** Glycolysis and gluconeogenesis (Wikipathways) in FC and **(B)** glycolysis (Hallmark) in TC **(C)** The green curve denotes the running ES for the gene set as the analysis moves down the ranked list of genes. Positive correlation, NES >0; Negative correlation, NES <0. Adj. P, adjusted p-value. These callouts also apply to the **Supplementary Figure 1**. **(D)** The 28 core glycolysis genes. Cells in red denote the genes identified as leading edge genes in these brain regions. **(E)** AD samples reveal lower glycolysis indices than that in the control in different brain regions. ***p<0.001. **(F)** The trends of glycolysis index in different Braak NFT stages using the GSE84422 dataset. Blue dots represent the individual samples. NES, Normalized enrichment score; GSEA, gene set enrichment analysis; AD, Alzheimer’s disease; FC, frontal cortex; TC, temporal cortex; ES, enrichment score; NFT, neurofibrillary tangles; and KEGG, Kyoto Encyclopedia of Genes and Genomes.

To investigate the clinical relevance of glycolysis in AD, we initially selected seven glycolysis-related gene sets from MSigDB, some of which have been previously considered. GSEA was performed on AD vs. control brain samples in expression profiles of four brain regions, namely the FC, TC, HP, and EC, from AlzData datasets using the aforementioned gene sets. The majority of glycolysis gene sets were significantly downregulated in at least one brain region in AD samples, compared with control brain tissues (**Figures 1A–C** and **Supplementary Figure 1**). For a downregulated gene set, the leading edge subset consisted of the genes ranked after the trough in the figure, which were the core genes causing the downregulation of this pathway. Thus, we filtered the genes identified as the GSEA leading-edge subsets in at least two brain regions (**Figure 1D**). The above core glycolysis genes were pooled to constitute a novel gene set of 28 genes, which was used to perform ssGSEA for calculating the ES as the glycolysis index.

To observe the clinical relevance of the glycolysis index, we initially applied this algorithm to the control and AD samples in four datasets from AlzData. The glycolysis indices were significantly lower in the AD group than that in the control group in all brain regions (**Figure 1E**). Subsequently, we used multivariate logistic regression models to predict AD with glycolysis index, age, sex, different brain regions, Aβ precursor protein (APP), microtubule associated protein tau (MAPT), and Presenilin 1/2 (PSEN1/2) gene expression as covariates. The glycolysis index still had significant results after adjusting the effects of the above covariates (**Supplementary Table S1**). Furthermore, using microarray data (GSE84422) from the GEO dataset as a validation dataset, we evaluated the prognostic value of the glycolysis index for patients with AD. Principal component analysis was performed on the combined expression profiles of 3 datasets. The results showed that the samples using different probe sets can be distinguished by the first principal component alone (**Supplementary Figure 2**). In 1991, Braak and Braak proposed Braak NFT stages, which were based on the distribution and severity of neurofibrillary tangles and neuropil threads (41). We analyzed the correlations between the glycolysis index and different Braak NFT stages. The glycolysis index decreased with higher stages of AD (**Figure 1F**). Eventually, the Spearman’s test was used to analyze the correlation between glycolysis indices and Braak stages in different brain regions, respectively. The results of the middle temporal gyrus, frontal pole and HP are significant in two probe sets (**Supplementary Figure 3**). Remarkably, we compared the ability of the glycolysis index and 7 glycolysis-related gene sets to distinguish between AD and controls, and the correlation with Braak stages. The results showed that glycolysis index has the most significant ability to distinguish between AD and controls in three of the four brain regions and was significantly correlated with Braak stages in two of the three data sets (**Supplementary Table S2**). We also observed the correlation of glycolysis index with Neuritic Plaque Density, APP, MAPT and PSEN1/2. The neuritic plaque density was defined as the average of neuritic plaque densities measured in five regions, including middle frontal gyrus, orbital frontal cortex, superior temporal gyrus, inferior parietal lobule, and occipital cortex. There was no or a weak negative correlation between the glycolysis index and Neuritic Plaque Density (**Supplementary Figure 4A**). Considering that they both are correlated with AD and Braak stages, they may be independent AD markers, respectively. Due to the lack of corresponding clinical data, the expression levels of APP and MAPT genes were used to represent the levels of Aβ42 and phospho tau (42). The expression levels of two other AD markers, PSEN1/2, were also included in the study. The results showed that these 4 genes were significantly correlated with glycolysis index in at least one brain region (**Supplementary Figures 4B–E**). In other words, the glycolysis index may serve as a reliable prognostic indicator in patients with AD.

### Screening of glycolysis-related genes and pathways in AD

**Figure 2:**
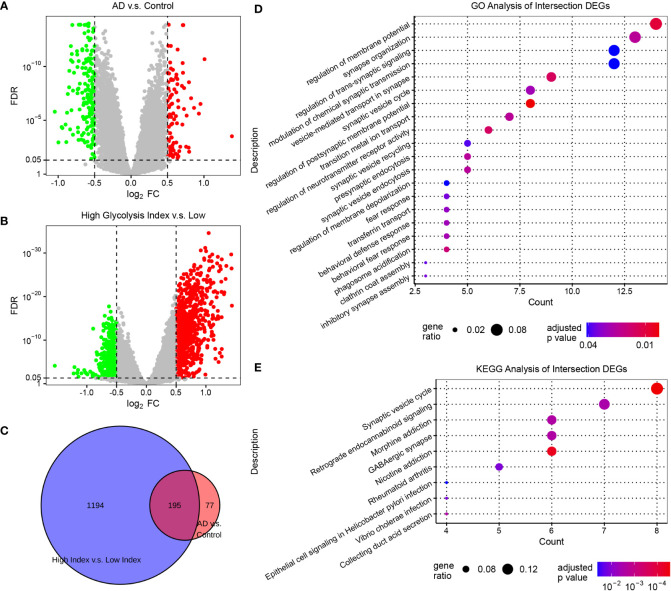
Screening glycolysis-related genes, and their relevant signaling pathways and functions in AD **(A)** Volcano plots of DEGs in AD vs. control samples. **(B)** Volcano plots of DEGs between AD samples with low and high glycolysis indices. The genes with FDR <0.05 and |log_2_ FC|>0.5 are considered significant. **(C)** Venn diagram of the overlapping genes between the two differential expression analyses. **(D)** The GO enrichment analysis of intersection DEGs. **(E)** The KEGG enrichment analysis of intersection DEGs. DEG, differentially expressed genes; AD, Alzheimer’s disease; FDR, false discovery rate; GO, gene ontology; and KEGG, Kyoto Encyclopedia of Genes and Genomes.

To characterize the transcriptomics of glycolysis-related genes in AD, we performed the differential expression analysis of the expression profiles from AlzData. The brain region, age, and sex were set as the covariates to exclude their influence. Subsequently, we screened 272 DEGs (FDR<0.05, |log2 FC|>0.5) between normal and AD brain tissues, and 1,389 DEGs (FDR<0.05, |log2 FC|>0.5) between AD samples with low and high glycolysis indices (**Figure 2A, B** and **Supplementary Table S3**). These 195 overlapping genes served as glycolysis-related DEGs in AD (**Figure 2C**). Furthermore, to explore the signaling pathways involved in glycolysis-related DEGs in AD, we conducted GO and KEGG enrichment analyses of the glycolysis-related DEGs. These genes were principally associated with the functions of the nervous system, including the regulation and transmission of synaptic and membrane potentials (**Figure 2D**). In addition, the KEGG enrichment analysis revealed that the signaling pathways of these genes were predominantly composed of the synaptic vesicle cycle, retrograde endocannabinoid signaling, and GABAergic synapses (**Figure 2E**). Thus, glycolysis-related DEGs play an important role in the neurodegenerative process of AD pathogenesis. Notably, we detected some immune-related pathways, such as rheumatoid arthritis, thus raising the question of the association between glycolysis and the brain microenvironment in AD.

### The role of glycolysis in the brain microenvironment of AD

**Figure 3:**
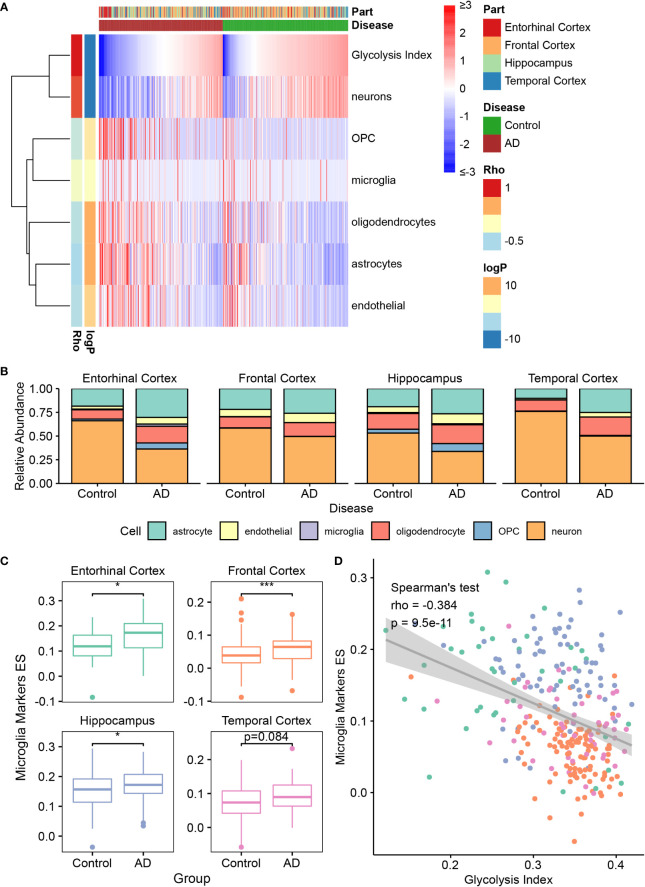
The association between glycolysis and the brain microenvironment **(A)** Heatmap depicts the abundance of six brain cells calculated by CIBERSORTx in AD and control samples. Rho: the Spearman’s rho between the abundance and glycolysis index. The significance of the differences between AD and control are measured by the directed log P values of the Wilcoxon’s rank-sum tests. A positive value denotes higher abundance in the AD group. **(B)** Relative abundance of six brain cell types in different brain regions between control and AD samples. **(C)** The quantification of microglia using the ES of microglia marker genes in different brain regions. **(D)** Spearman’s correlation analysis between the glycolysis index and the ES of microglia markers. Different colors indicate different regions, and it is the same as the color in **(C)**. *p<0.05, ***p<0.001. AD, Alzheimer’s disease; ES, enrichment score.

According to previous studies, almost all cell types in the brain, such as neurons, astrocytes, microglia, oligodendrocytes, and endothelial cells, play important roles in AD development (43–47). Therefore, we investigated the correlation between glycolysis and different cell types in the AD brain microenvironment. First, we estimated the abundance of immune cells in AlzData samples using the following decomposition tools: EPIC, ImmuCellAI, and CIBERSORTx (36–38) (**Figure 3A** and **Supplementary Figure 5**). However, these tools were designed for estimating the tumor microenvironment by default; nonetheless, the cells in the tumor microenvironment were not similar to those in the brain. For example, microglia are the resident macrophages of the brain but are not exactly identical to macrophages in the tumor. To obtain the cell-type content estimates closer to the true situation in the brain, we used CIBERSORTx in the custom cell-type mode, which can build a personalized model from brain single-cell expression profiles for deconvolution. To this end, we used a brain single-cell RNA-seq dataset from GEO datasets containing astrocytes, endothelial cells, microglia, oligodendrocytes, OPCs, and neurons. In a follow-up study, we principally focused on the estimation of brain cells using the custom CIBERSORTx model. By applying Spearman’s correlation analysis, the glycolysis index was positively correlated with cell subtypes, including neurons, follicular helper T cells, activated natural killer cells, cluster of differentiation 8 T cells, cluster of differentiation 4 T cells, and T helper type 1 cells. By contrast, it was negatively correlated with microglia, macrophages, astrocytes, OPC, endothelial cells, oligodendrocytes, and B cells. Meanwhile, the relative abundance of brain cells negatively correlated with the glycolysis index, including astrocytes, endothelial cells, microglia, oligodendrocytes, and OPC was higher in AD, and vice versa (e.g., neurons) (**Figure 3A, B** and **Supplementary Figure 6**). These results were also significant upon excluding the effects of different brain regions using ANOVA or a linear model, and on using the absolute abundance instead of relative abundance. Subsequently, we focused on the relationship between glycolysis and microglia in AD. To confirm the findings based on the custom CIBERSORTx model, we collected the cell markers of microglia from the CellMarker (34) database, and calculated the ES of these marker genes in AD and control data using ssGSEA. Similar to the above phenomena, the expression of microglial markers was significantly higher in the EC, FC, and HP of AD, and negatively correlated with the glycolysis index (**Figures 3C, D**). In summary, these results show that glycolysis plays a vital role in the AD brain microenvironment.

### Glycolysis-related brain cell markers revealed the potential relationship between glycolysis and the brain microenvironment of AD

**Figure 4:**
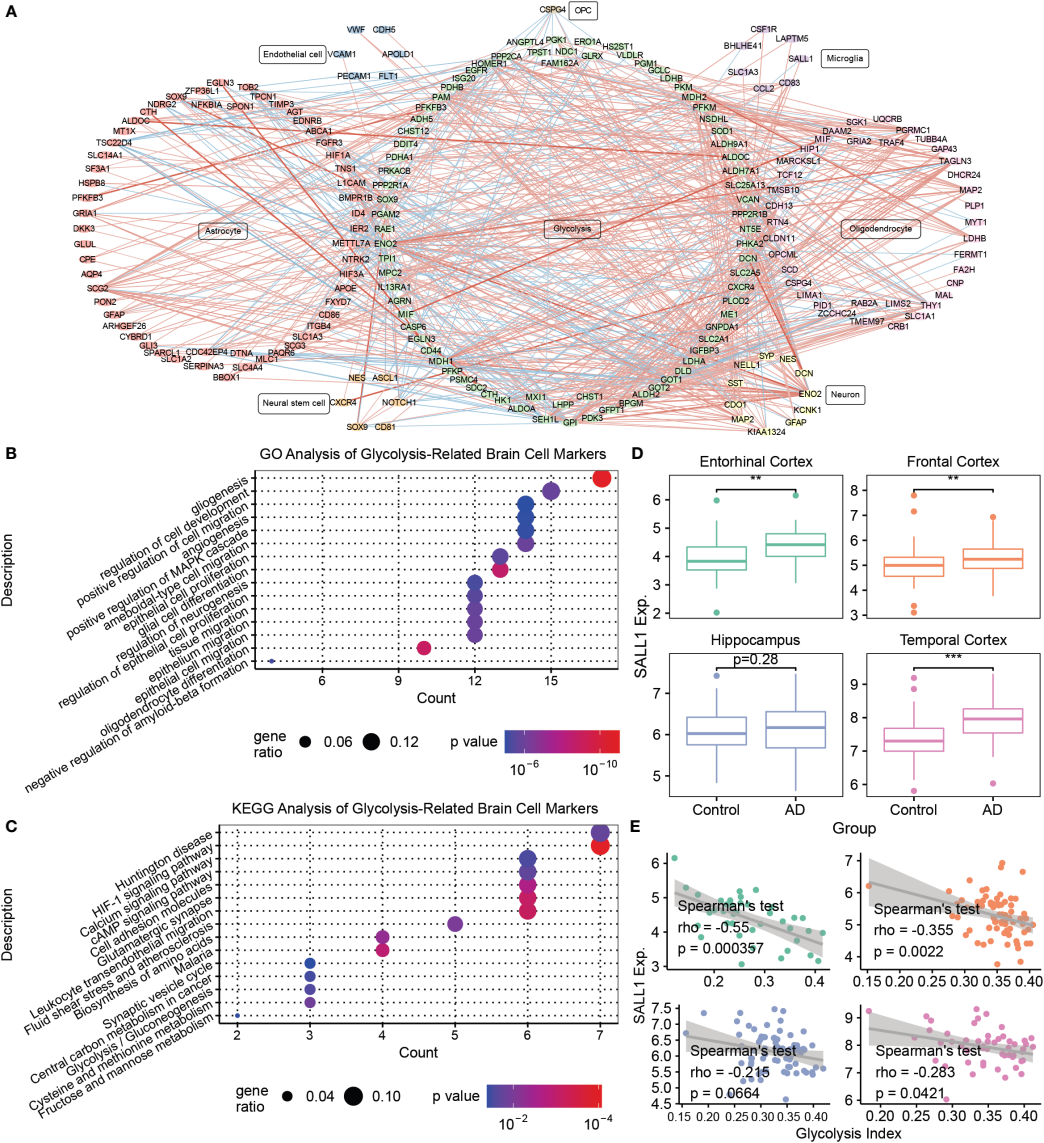
Mining of glycolysis-related brain cell markers **(A)** The correlations between glycolysis-related genes and various brain cell marker genes. The thickness of the line denotes the number of metrics supporting the correlation. The red line denotes a positive correlation, whereas the blue line denotes a negative correlation. **(B)** The GO enrichment analysis of glycolysis-related brain cell markers. **(C)** The KEGG enrichment analysis of glycolysis-related brain cell markers. **(D)** Boxplots of SALL1 expression in different brain regions between the control and AD samples. **(E)** Spearman’s correlation analysis between the glycolysis index and SALL1 expression. **p<0.01, ***p<0.001. AD, Alzheimer’s disease; GO, gene ontology; SALL1, Spalt Like Transcription Factor 1; and KEGG, Kyoto Encyclopedia of Genes and Genomes.

To further explore the relationship between glycolysis and the brain microenvironment in AD, we conducted a co-expression analysis and PPI analysis between the genes from seven glycolysis-related gene sets and marker genes of seven brain cells (astrocytes, endothelial cells, microglia, neural stem cells, neurons, oligodendrocytes, and OPC). Consequently, we constructed a glycolysis-brain-cell gene connection network (**Figure 4A** and **Supplementary Table S4**). Moreover, we used glycolysis-related brain cell markers in AD to perform GO and KEGG analyses (**Figures 4B, C**). Specifically, as brain cell markers, these genes were highly correlated with the regulation of the development of several types of brain cells, such as endothelial cells and oligodendrocytes. In addition, some of the genes play roles in the negative regulation of Aβ formation, consistent with our findings that AD brains display a downregulation in glycolysis. In addition, the mitogen-activated protein kinase (MAPK) signaling pathway was significantly enriched. A previous study demonstrated that the inhibition of p38α MAPK expression could promote beta-site APP cleaving enzyme 1 degradation and reduce neuronal Aβ generation in AD (48). Moreover, P38α MAPK is considered a promising target for AD therapy (49). Hence, our findings illustrated the complex interactions between glycolysis and the brain microenvironment of AD, and corroborated previously described results. We focused on microglial markers to better understand the relationship between glycolysis and the immune microenvironment in patients with AD. For example, SALL1, the microglia marker gene, was higher in AD brains than that in the control, besides being negatively correlated with the glycolysis index in the EC, FC, and TC (**Figures 4D, E**). Remarkably, we also performed the same analysis for activated microglia markers (50). Similar to SALL1, the results showed that most activated microglia markers were also higher in the AD brain than in the control, and negatively correlated with glycolysis index. Neither had significant opposite results that activated microglia markers were lower in the AD brain or positively correlated with glycolysis index (**Supplementary Table S5**). Thus, glycolysis may influence the brain microenvironment of AD in multiple ways, and a series of target biomarkers may play important roles in basic scientific research and drug design.

### Validation of glycolysis-related microglia markers in the APP/PS1 AD model

**Figure 5:**
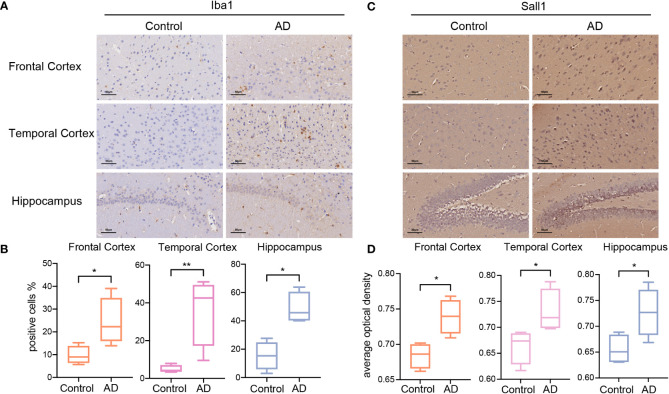
The expression of Iba1 and Sall1 in C57BL/6J mice and APP/PS1 AD model mice **(A, C)** IHC staining for Iba1 and Sall1 in different regions between the control and AD samples. **(B, D)** The expression levels of Iba1 and Sall1 in different regions between the control and AD samples, n=4. *p<0.05, **p<0.01. AD, Alzheimer’s disease; SALL1, Spalt Like Transcription Factor 1; and Iba1, ionized calcium-binding adapter molecule 1.

To validate the results of bioinformatic analyses, we investigated the abundance of microglia and the expression of Sall1, a glycolysis-related microglia marker, in the AD model and control group. APP/PS1 mice were previously reported as an ideal model for AD and have a C57BL/6J background; thus, we selected APP/PS1 and C57BL/6J mice as our research objects (51, 52). First, using IHC, we detected Iba1 expression levels in different brain regions of APP/PS1 AD and C57BL/6J mice. Iba1 is a common marker for microglia; its expression levels were significantly higher in the FC, TC, and HP of AD, thereby indicating greater infiltrated microglia in different AD brain regions (**Figures 5A, B**). Second, we detected Sall1 expression levels in different brain regions to determine the expression level of glycolysis-related microglial markers at the protein level. Compared with the control group, Sall1 expression levels were significantly increased in the FC, TC, and HP in AD mice (**Figures 5C, D**). Thus, there were greater infiltrated microglia and higher expression of Sall1 in the APP/PS1 AD model than that in the control group, consistent with our aforementioned analyses.

### The predictive value of glycolysis index in normal samples

**Figure 6:**
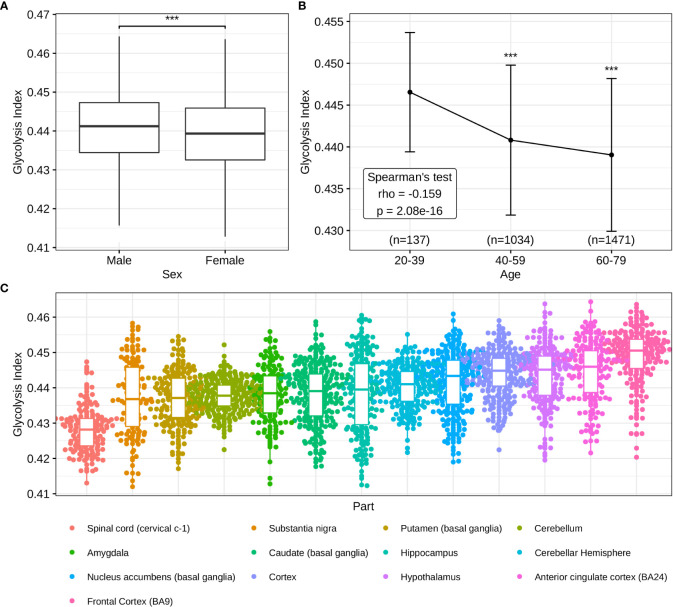
Distributions of glycolysis indices in normal brain tissues **(A)** The sex difference of glycolysis indices. **(B)** Glycolysis indices in different age groups. **(C)** Distributions of glycolysis indices in the different parts of the brain. ***p<0.001.

In addition to the pathological significance of the glycolysis index, we investigated its predictive value in normal brains. In contrast to males, lower glycolysis indices were observed in females (**Figure 6A**), consistent with previous studies demonstrating a higher incidence of AD in women (53). The Spearman’s test of the glycolysis index in different age groups indicated that the glycolysis index decreased with age, and was lowest in the most common age of AD (**Figure 6B**). Eventually, different brain regions presented a diverse distribution of the glycolysis indices (**Figure 6C**), thus suggesting brain regions with lower indices may pose a higher risk of AD and require greater attention in future studies. These results confirmed the clinical and predictive value of the glycolysis index in AD.

## Discussion

AD was first proposed by the German neuropathologist Alois Alzheimer in 1906, and researchers have discovered a spectrum of neuropathological manifestations (54, 55). It is challenging to identify patients with AD at an early stage, and currently there is no effective cure (56). Instead, several patients have to receive palliative care. Researchers have widely studied and proven the mechanism of Aβ; nonetheless, vaccines and elimination targeting Aβ do not successfully prevent AD progression (57, 58). This necessitates exploring the pathogenesis of AD for early diagnosis. Current research actively focuses on identifying potential novel therapeutic targets for AD (59), whereas the mechanism of impaired glucose metabolism provides new insights into this field.

In this study, we observed downregulated glycolytic processes in four different brain regions in AD models. Impaired glucose metabolism may contribute to neuronal degeneration and cognitive impairment in patients with AD (11, 60–62). Interestingly, decreased glycolysis presumably decelerates the aging process but plays a pathogenic role in AD (63). This could be attributed to TPI dysfunction and mutations, which is one of the regulatory enzymes in glycolysis. TPI converts dihydroxyacetone phosphate (DHAP) to glyceraldehyde-3-phosphate (GAP), following which its structure changes, thus resulting in decreased activity (64). This leads to reduced GAP production and the increased accumulation of DHAP. Reduced GAP production causes the underproduction of NADH, which results in mitochondrial dysfunction in AD; meanwhile, increased DHAP accumulation contributes to the accumulation of advanced glycation end products (AGEs), which stimulate Aβ aggregation and affect the cognitive functions in patients with AD (65–67). Impaired glucose metabolism can induce various pathophysiological cascades, such as Aβ accumulation, tau hyperphosphorylation, inflammation, AGEs, and excitotoxicity (68). The detailed mechanism requires further elucidation; however, the 28 core glycolysis genes may play a key role in the glycolysis-related pathogenesis of AD. A study from Australia demonstrated that the suppression of the dihydrolipoamide dehydrogenase gene could inhibit Aβ pathogenicity and may be simultaneously considered a potential therapeutic target (69). Previous studies applied the striatal amyloid plaque density to predict Braak NFT stages in AD (70). Currently, there are no studies on the prediction of Braak NFT stages in AD using indicators of glycolysis, and we used the glycolysis index to identify the Braak NFT stages in AD. Our study quantified the level of glucose metabolism using the glycolysis index and demonstrated its clinical relevance and prognostic significance in AD.

Several cell types in the brain are intimately involved in AD pathogenesis and glycolysis. Impaired astrocytic glycolysis decreases L-serine, thus leading to cognitive impairment in AD (71). Moreover, oligodendrocyte glycolytic deficiency through the dynamin-related protein 1- hexokinase 1- NLR family pyrin domain containing 3 signaling axis contributes to white matter degeneration and cognitive impairment in AD (72). Microglial activation was induced by Aβ, followed by the secretion of pro-inflammatory cytokines, which changed into an innate immune-tolerant state within 5 days, thus exhibiting glycolysis impairment (73). Herein, we examined the connection between glycolysis and seven brain cell types to investigate the underlying mechanism in the AD brain. The resulting network systematically clarified this relationship at the gene level, which could explain the functioning of the glycolytic process in the brain microenvironment of AD. For example, the glycolysis-related gene SEH1-like is required for oligodendrocyte differentiation, myelination, and post-injury remyelination in the central nervous system (74). In another study, MIF, an oligodendrocyte-related marker, led to a higher rate of glycolysis (75). Decreased activities of thiamine-dependent enzymes exist in AD, which contributes to the dysfunction of glucose metabolic and inflammatory processes (76–78). We investigated the relationship between glycolysis and the immune microenvironment, focusing on seven microglial markers. Microglia are an important part of the brain’s inflammatory response and play a role in the phagocytosis of Aβ plaques in AD (79). There are several reports on the role of microglia in AD (80–82). We screened seven key microglial markers, most of which were related to neurodegenerative diseases. Chemokine (C-C motif) ligand 2 (CCL2) may be involved in the pathways recruiting microglia in chronic traumatic encephalopathy, a progressive neurodegenerative disease. In addition, the negative correlation with Aβ42 in males suggests that higher CCL2 recruits more microglia to phagocytose the plaques (83). Joly-Amado et al. revealed that CCL2 overexpression promotes an increase in pathogenic tau and harmful glial inflammation (84). In addition, inhibitors targeting receptor of the colony-stimulating factor-1 activity can reduce microglial proliferation and neurodegeneration, slow neuronal damage and disease progression, and prevent cognitive decline in AD (85–87). Beschorner et al. confirmed that the expression of excitatory amino acid transporter 1 (also termed SLC1A3) in activated microglia reflects a potential neuroprotective function (88). Remarkably, one study revealed that inhibition of SALL1 could reduce the level of CDH1 (89). The other research showed that upregulation of CDH1 could inhibits 6-phosphofructo-2-kinase/fructose-2,6-bisphosphatase isoform 3, and further inhibit glycolysis (90). These findings indicate a close relationship between glycolysis and the brain microenvironment in AD, thereby offering novel drug targets for future therapies.

In summary, our results demonstrated the clinical significance of the glycolysis index and elucidated potential molecular connectors that linked the brain microenvironment and glycolysis in AD. Glycolysis-related genes displayed broad interactions with abundant brain cell markers, which may play a crucial role in AD brain function. Furthermore, we not only revealed the clinical relevance and excellent prognostic value of the glycolysis index using high-throughput transcriptomic data but also validated the expression of glycolysis-related microglia marker Sall1 in APP/PS1 mice. Essentially, our findings provided novel insights into AD pathogenesis and may provide potential novel therapeutic targets for its treatment. However, several limitations of this study should be noted. First, the influence of chronic diseases, smoking, and other factors could not be excluded due to the lack of corresponding clinical data. Second, the glycolysis index was validated in 3 sub-datasets of one AD cohort. The results need to be further validated in more AD cohorts. Finally, the function and the exact mechanism and pathway of SALL1 in glycolysis need to be further investigated using biochemical experiments.

## Data availability statement

Publicly available datasets were analyzed in this study. This data can be found in the AlzData, GTEx, MSigDB and CellMarker repositories.

## Ethics statement

The animal study was reviewed and approved by Animal Experiments and Experimental Animal Welfare Committee of Capital Medical University.

## Author contributions

Conception and design of the research: ZQ, YA. Acquisition of data: ZQ, XW, XH, and FJ. Analysis and interpretation of the data: XW, XB, and XJ. Statistical analysis: ZQ, XB, and XJ. Writing of the manuscript: ZQ, DW. Critical revision of the manuscript for intellectual content: YA. All authors have read and approved the final draft. All authors contributed to the article and approved the submitted version.

## Acknowledgments

We thank the investigators, volunteers, and patients who participated in the GEO for providing data.

## Conflict of interest

Author XH, DW and FJ are employed by Beijing Yihua Biotechnology Co., Ltd.

The remaining authors declare that the research was conducted in the absence of any commercial or financial relationships that could be construed as a potential conflict of interest.

## Publisher’s note

All claims expressed in this article are solely those of the authors and do not necessarily represent those of their affiliated organizations, or those of the publisher, the editors and the reviewers. Any product that may be evaluated in this article, or claim that may be made by its manufacturer, is not guaranteed or endorsed by the publisher.
