# Supplementary material for: The significance of glycolysis index and its correlations with immune infiltrates in Alzheimer’s disease
Source: Front Immunol. 2022 Oct 24;13:960906. doi: 10.3389/fimmu.2022.960906 (PMC9637950; doi:10.3389/fimmu.2022.960906)
Supplement: Supplementary file 1 [file DataSheet_1.docx]

Supplementary Material

## Supplementary Figures


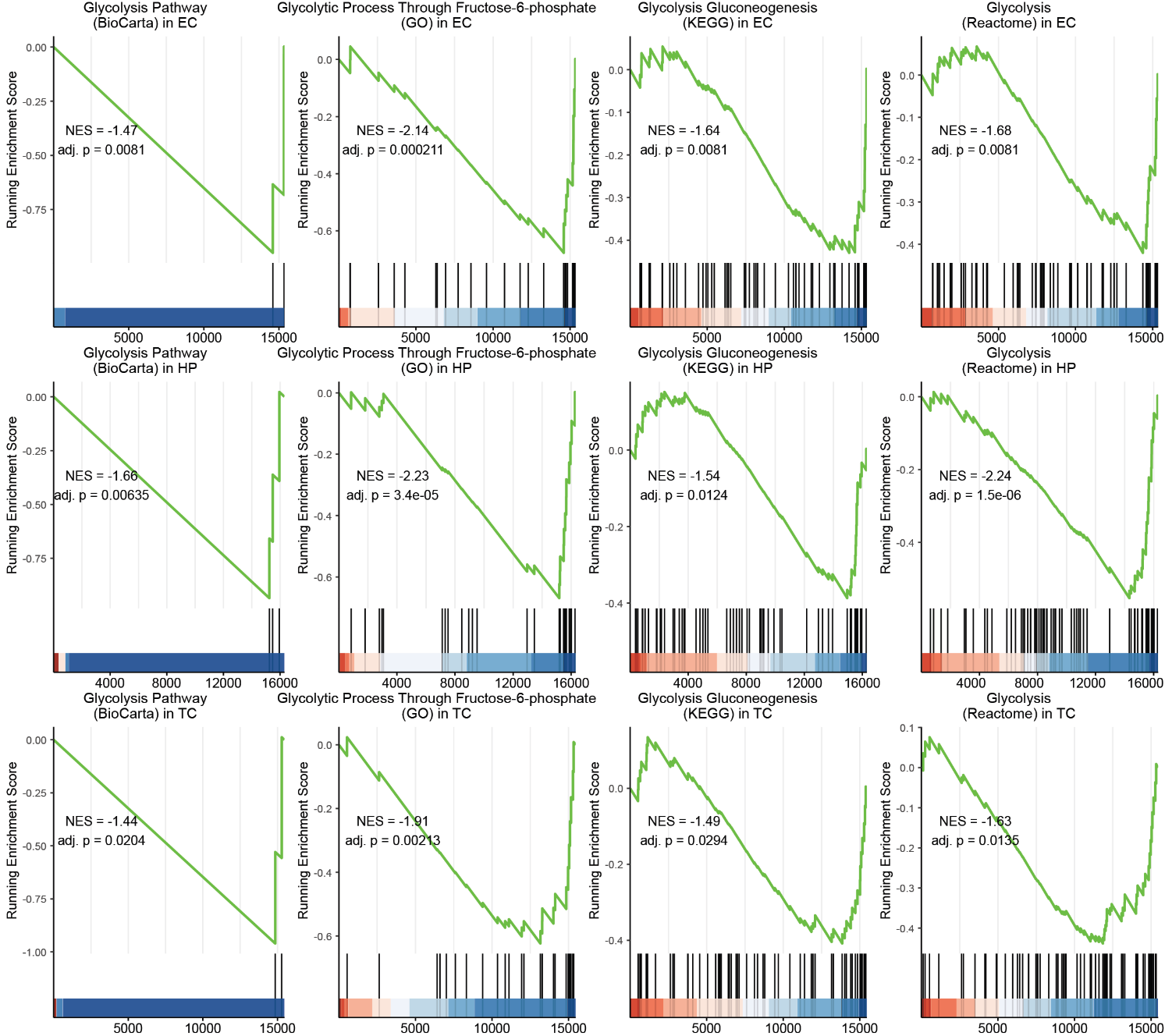


**Supplementary Figure 1.** An enrichment analysis of four glycolysis-related gene sets of BioCarta, GO, KEGG, and Reactome in AD vs. control samples of three different brain regions (EC, HP, and TC).

AD, Alzheimer’s disease; GO, gene ontology; KEGG, Kyoto Encyclopedia of Genes and Genomes; TC, temporal cortex; HP, hippocampus, and EC, entorhinal cortex.


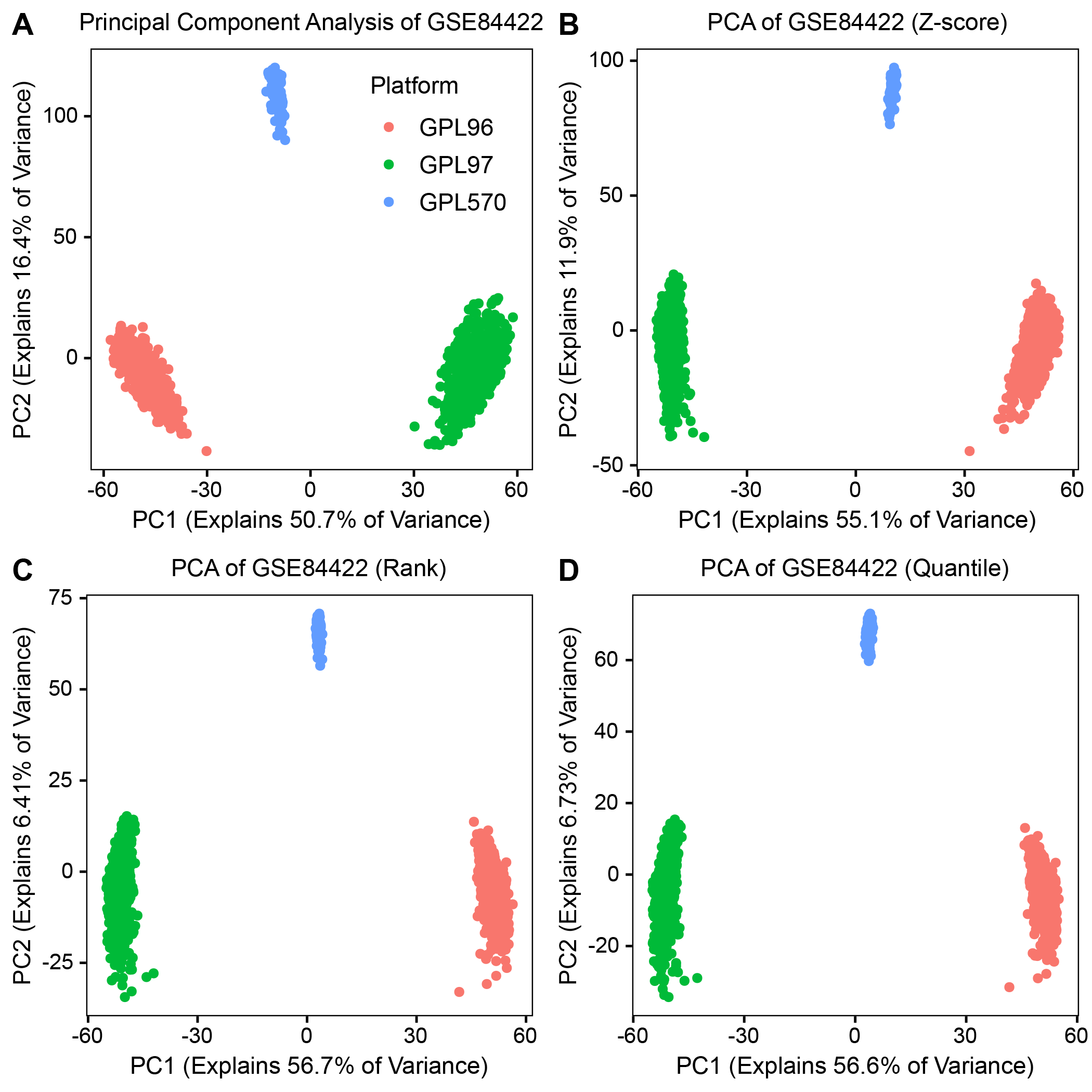


**Supplementary Figure 2.** Principal component analyses on the combined expression profiles of 3 datasets of GSE84422.


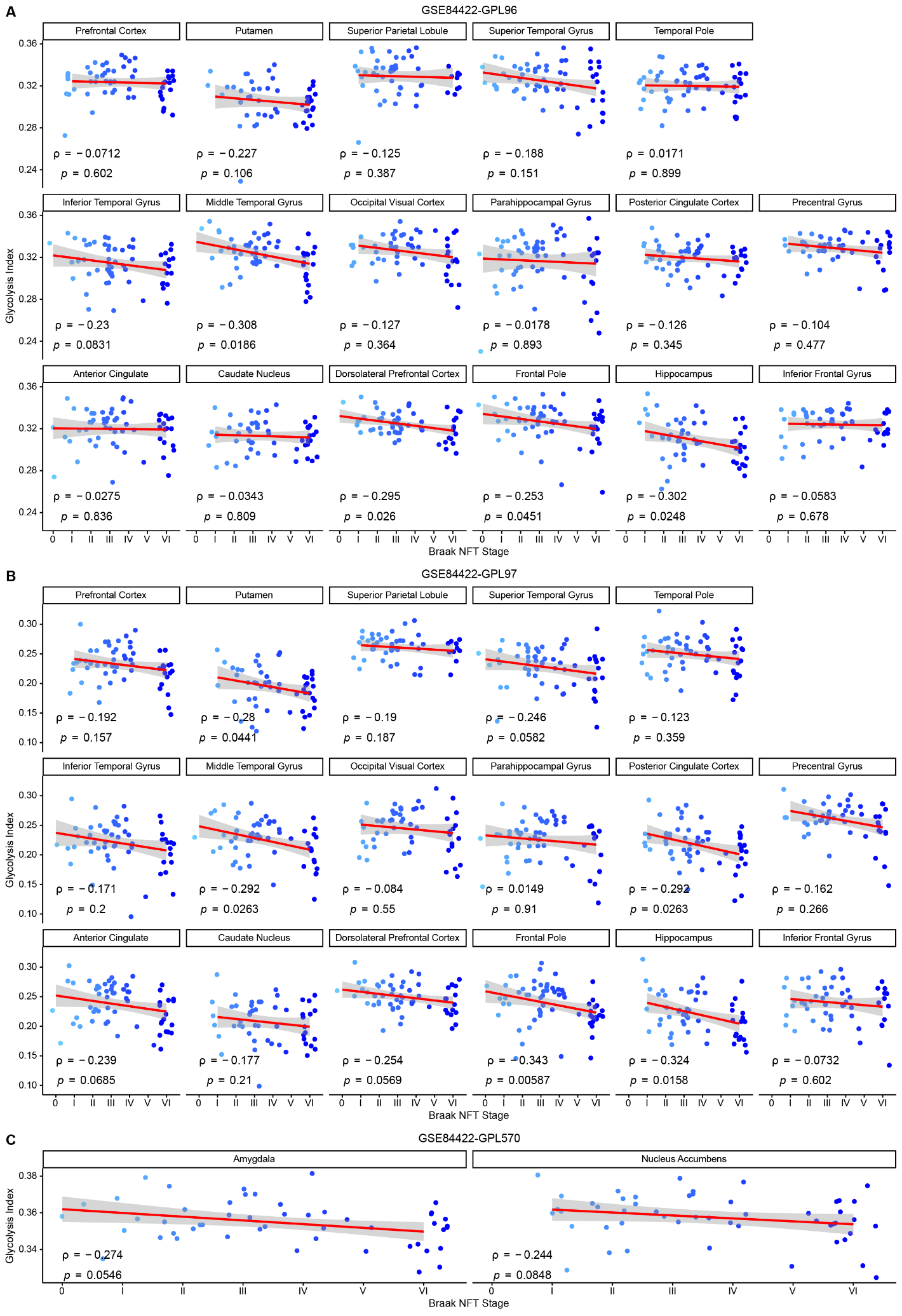


**Supplementary Figure 3.** Spearman’s correlation analyses between the glycolysis indices and Braak stages in different brain regions using GSE84422.


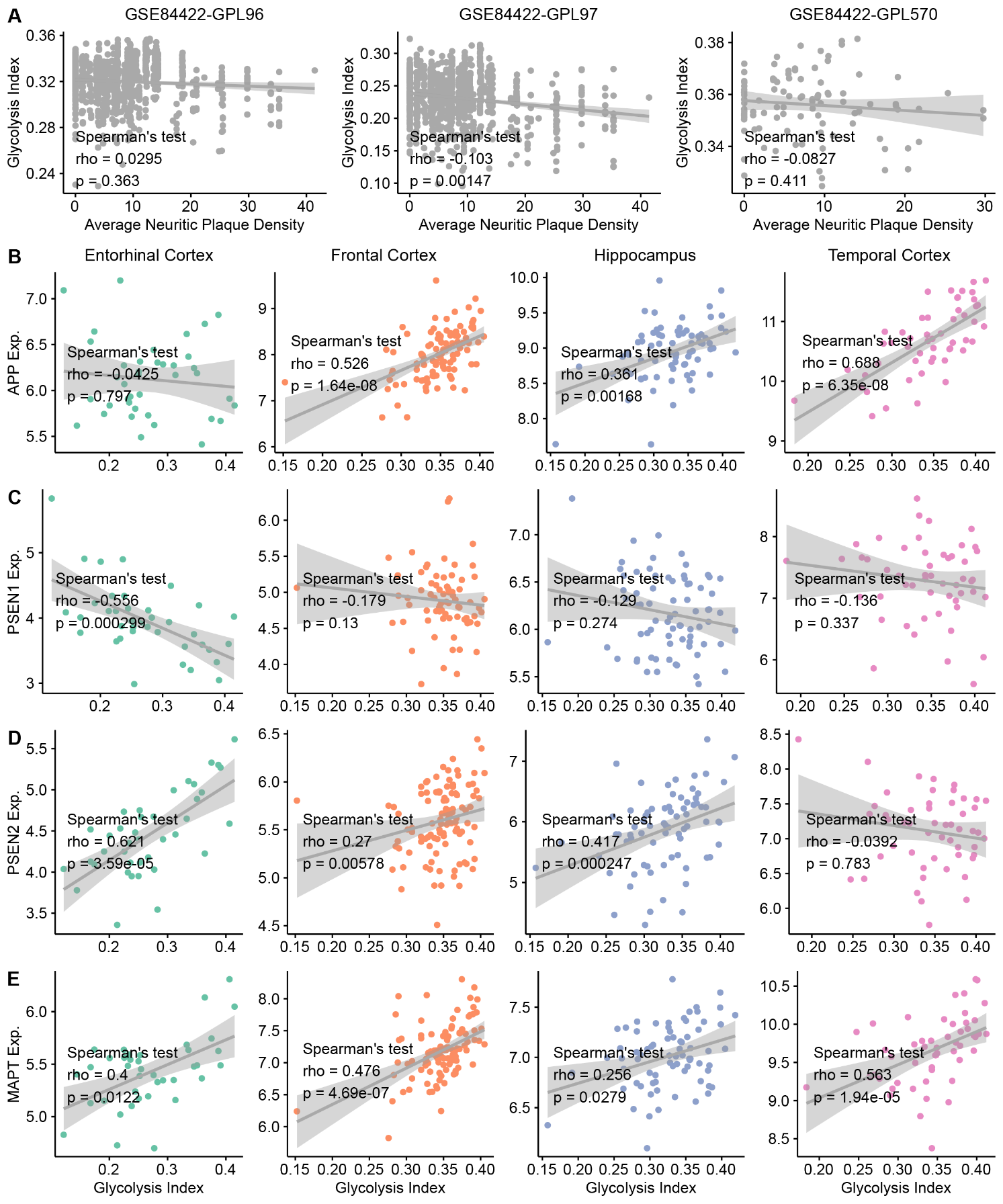


**Supplementary Figure 4.** (A) Correlation between glycolysis index and average Neuritic Plaque Density. (B-E) Spearman’s correlation analysis between the glycolysis index and the expression of 4 AD markers.

AD, Alzheimer’s disease; APP, Aβ precursor protein; PSEN1/2, Presenilin 1/2, and MAPT, microtubule associated protein tau.


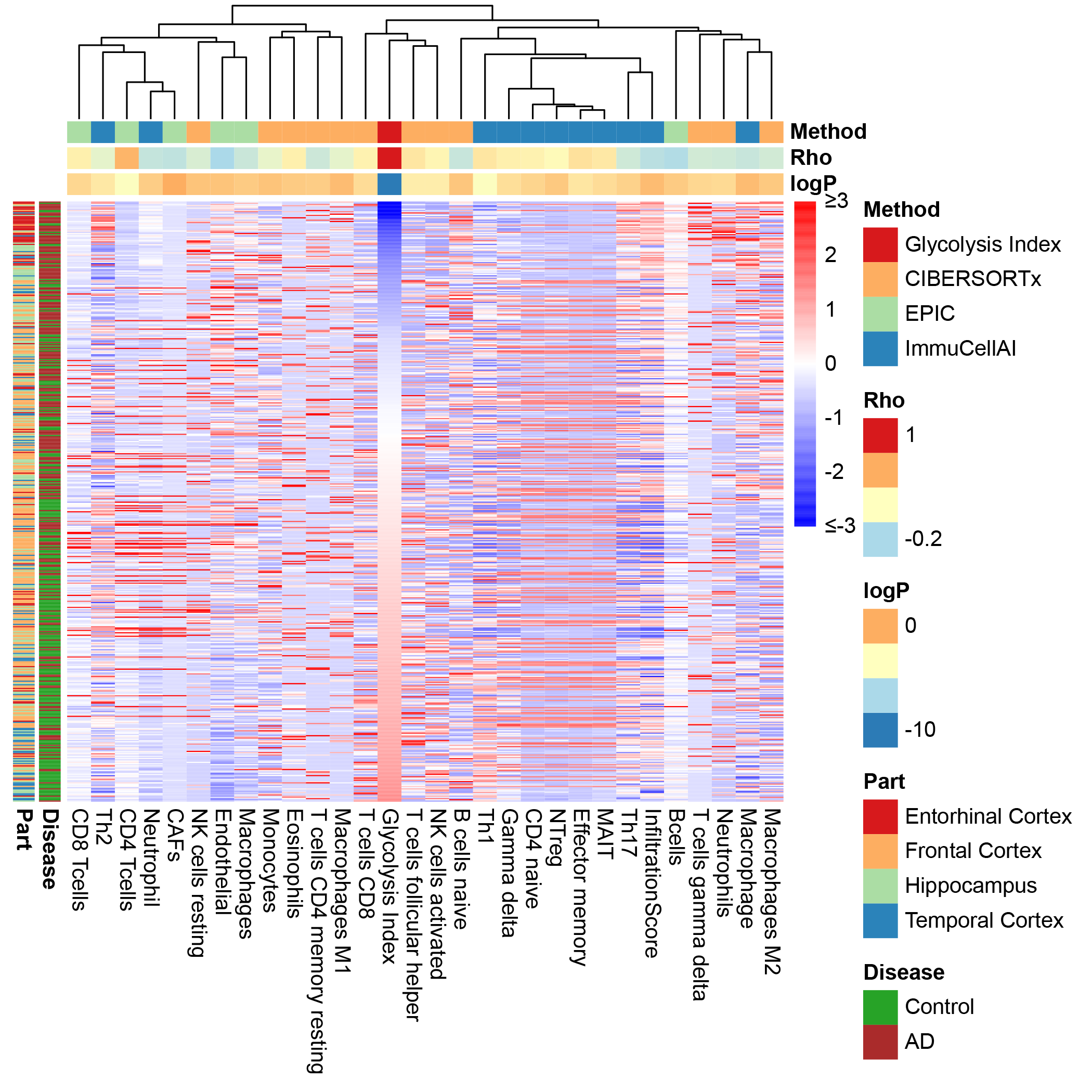


**Supplementary Figure 5.** Heatmap depicts the abundance of various immune cell types calculated by different methods in AD and control samples of four brain regions. Statistical significance assessed in the same way as for Figure 3A.

AD, Alzheimer’s disease.

**
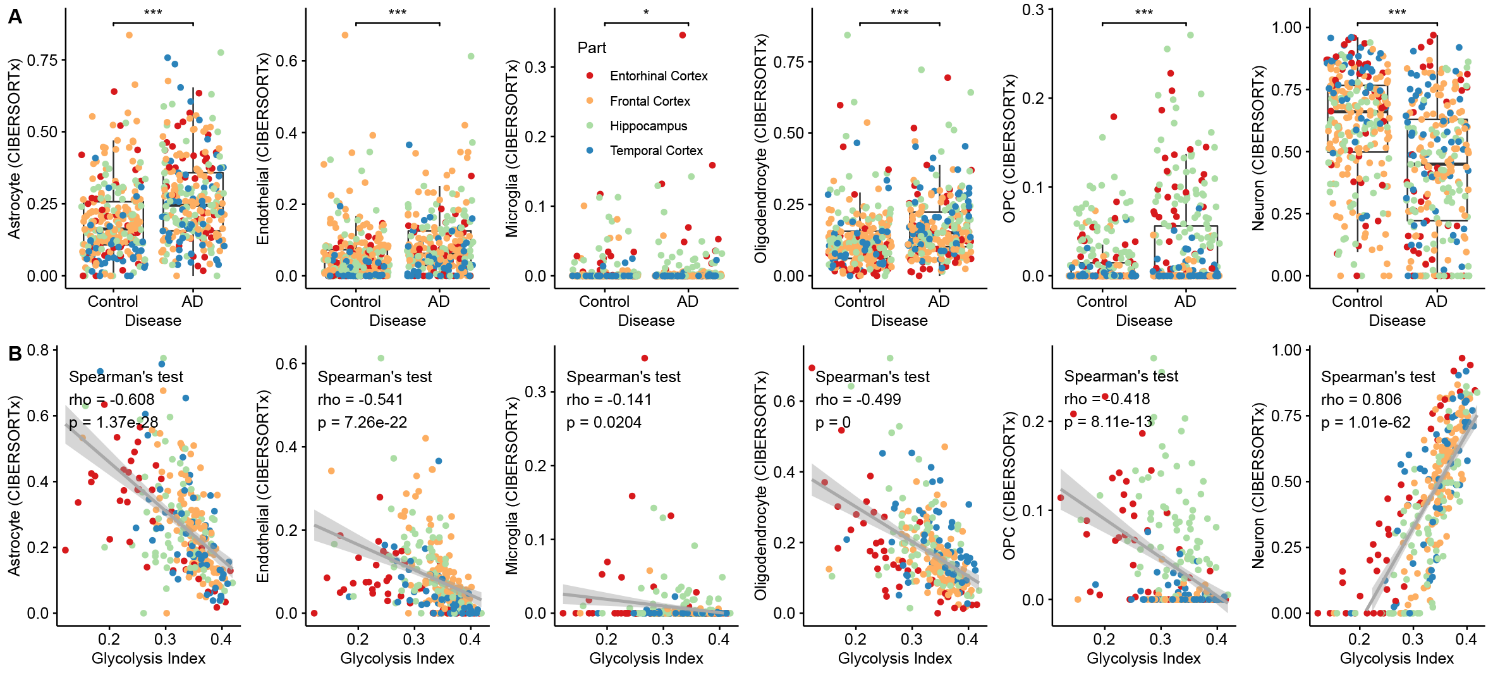
**

**Supplementary Figure 6.** (A) Differences in the abundance of various brain cell types between the control and AD samples. (B) Correlations between cell abundance and the glycolysis index.

AD, Alzheimer’s disease.
